# Role of homocysteine, vitamins B12, B6, B1, and folate in Saudi patients with premature acute coronary syndrome

**DOI:** 10.3389/fmed.2026.1847202

**Published:** 2026-07-10

**Authors:** Khalid Alfaraidy, Ayman Mohamed, Alexander Woodman, Rehab Al-Ansari, Yasser A. Al Malki, Fatimah S. Alayidh, Yaser Alnaam, Abdulrahman Al Husil, Mohamed I. Amin, Masniwati Sigem, Yousef M. Hawsawi, Lamiaa H. Al-Jamea

**Affiliations:** 1 Department of Cardiology, King Fahad Military Medical Complex, Ministry of Defense Health Services, Dhahran, Saudi Arabia; 2 Department of Scientific Research, King Fahad Military Medical Complex, Ministry of Defense Health Services, Dhahran, Saudi Arabia; 3 Adult Hematology Unit, Department of Internal Medicine, King Fahad Military Medical Complex, Ministry of Defense Health Services, Dhahran, Saudi Arabia; 4 Department of Medical Laboratory, King Fahad Military Medical Complex, Ministry of Defense Health Services, Dhahran, Saudi Arabia; 5 Department of Clinical Laboratory Sciences, Prince Sultan Military College of Health Sciences, Dammam, Saudi Arabia; 6 Research Center, King Faisal Specialist Hospital and Research Center, Jeddah, Saudi Arabia; 7 Department of Biochemistry and Molecular Medicine, College of Medicine, Alfaisal University, Riyadh, Saudi Arabia; 8 Department of Academic Affairs and Training, King Fahad Military Medical Complex, Ministry of Defense Health Services, Dhahran, Saudi Arabia

**Keywords:** folate, homocysteine, premature acute coronary syndrome, premature coronary artery disease, Saudi Arabia, vitamin B1, vitamin B12, vitamin B6

## Abstract

Homocysteine (HCY) has been implicated in cardiovascular disease through adverse effects on vascular endothelium, oxidative stress, and thrombogenic pathways. Saudi Arabia has a young population and an increasing burden of cardiovascular disease, yet regional data on HCY and B-vitamin profiles in premature acute coronary syndrome (ACS) remain limited. This single-center cross-sectional exploratory study evaluated serum HCY, vitamins B12, B6, B1, and folate levels in 80 patients with premature ACS at King Fahd Military Medical Complex (KFMMC), Dhahran, Saudi Arabia. Premature ACS was defined as ACS occurring in men aged <=55 years and women aged <=65 years. Clinical, laboratory, electrocardiographic, echocardiographic, angiographic, and SYNTAX score II 2020 data were collected. Spearman correlation showed a weak but statistically significant inverse association between HCY and folate (rho = −0.254, *p* = 0.040; approximate R2 = 0.065), whereas correlations between HCY and vitamins B12, B1, and B6 were not statistically significant. Most patients had low SYNTAX score categories, and exploratory ordinal regression suggested associations between selected biomarkers/clinical variables and SYNTAX score category; however, these findings should be interpreted cautiously because of the small sample size, absence of a control group, and risk of model instability. The study provides preliminary descriptive data on HCY and B-vitamin status among Saudi patients with premature ACS and highlights the need for larger, controlled, multicenter studies incorporating dietary assessment and genetic determinants such as MTHFR polymorphisms.

## 1 Introduction

Coronary artery disease (CAD) is the most common type of heart disease worldwide and remains a major cause of morbidity and mortality. Although CAD is traditionally more frequent in older individuals, premature coronary artery disease (PCAD) is increasingly recognized as an important public health problem. Definitions of premature CAD vary across studies, with age thresholds typically ranging from 45 to 65 years. In the present study, premature ACS was defined as ACS occurring in men aged < =55 years and women aged < =65 years, consistent with the eligibility criteria used for this cohort. PCAD occurring at younger ages can lead to many years of lost productivity and a substantial healthcare burden (1–4).

Predisposing risk factors leading to PCAD are modifiable and non-modifiable; more than four-fifths of those with PCAD have at least one modifiable risk factor. For example, smoking, high blood pressure, diabetes, physical inactivity, obesity, dyslipidemia, and psychosocial stress. Non-modifiable risk factors include but are not limited to age, gender, ethnicity, family history of heart disease, and homocystinuria (5–7).

Homocystinuria is a rare genetic disorder characterized by significantly elevated serum homocysteine (HCY), an intermediate product of methionine metabolism. Homocysteine is also known to mediate cardiovascular problems through its adverse effects on cardiovascular endothelium and smooth muscle cells (8, 9). In patients with homocystinuria, hyperhomocysteinemia promotes the development of atherosclerotic lesions and is a cause of PCAD. The main mechanisms of the atherogenic action of HCY are considered to be oxidation of homocysteine-related LDL atherogenesis, inhibition of vascular endothelial growth combined with stimulation of smooth muscle cell proliferation, and interference with the coagulation and fibrinolytic systems. Previously, we reported several variants in Saudi populations that associated with several genetic conditions in Saudi population (10–12). Other rarer causes of severe hyperhomocysteinemia include homozygous MTHFR deficiency, methionine synthase deficiency, and methionine synthase deficiency due to genetic defects in vitamin B12 and vitamin B6 metabolism. The prevalence of hyperhomocysteinemia can vary significantly between populations and is likely to be influenced by age, sex, diet, and genetic background (8, 9, 13).

The Kingdom of Saudi Arabia, which has one of the youngest populations in the world, has witnessed rapid socio-economic transformation, urbanization, and lifestyle changes in recent decades, which has had a direct impact on the population's health, including high rates of cardiovascular diseases (CVD) (14–17). According to recent data, the overall prevalence of CVD was 1.6% across all regions of Saudi Arabia, 1.9% among men and 1.4% among women (12). The highest prevalence of CVD was recorded in the oldest age category of 65 years and above (11.1%), among men, and in the Makkah region (1.92%). Nevertheless, epidemiological data on PCAD in Saudi Arabia are limited (14–17).

To the best of the authors' knowledge, this is among the first studies in Saudi Arabia to describe HCY, vitamin B1, B6, B12, and folate levels in patients with premature ACS. The study was designed as an exploratory single-cohort analysis to characterize biomarker distributions and assess within-cohort associations with clinical and angiographic parameters, rather than to determine causality or predict the onset of ACS.

## 2 Methods

### 2.1 Study design and setting

This cross-sectional exploratory study was conducted among 80 patients with premature ACS at King Fahd Military Medical Complex (KFMMC), Dhahran, Saudi Arabia. Because all enrolled participants had established ACS, the study was not designed to estimate the risk of ACS onset or to infer causality between biomarkers and ACS development.

### 2.2 Study setting and sample

Initially, the study targeted a sample size of *n* = 214 patients across multiple centers prior to the lockdowns caused by COVID-19. However, due to restrictions, the study was reduced to one center, and a sample size of *n* = 80 patients was deemed feasible during that time.

Since this was the first such study in Saudi Arabia, the researchers referred to the data of Kumar et al. (18), who reported a prevalence of hyperhomocysteinemia of 83.3%. This prevalence value was applied to calculate the optimal sample size for the current study. Using the categorical sample size formula, the required sample size to detect a statistically significant result at a 95% confidence level and a margin of error of 0.50, assuming a two-tailed statistical test, was calculated as *n* = 214 patients.

The achieved sample size of *n* = 80 reflected logistical constraints during the COVID-19 lockdown period. The original sample size calculation was based on prevalence estimation and should be distinguished from the requirements for multivariable regression modeling. Therefore, multivariable findings in this study should be considered exploratory and hypothesis-generating, with limited statistical power and potential model instability.

### 2.3 Eligibility criteria

Inclusion criteria: Male patients aged < = 55 years and female patients aged < = 65 years presenting with their first episode of ACS and considered candidates for coronary angiography were eligible. ACS included ST-segment elevation myocardial infarction (STEMI), non-ST-segment elevation myocardial infarction (NSTEMI), and unstable angina.

Exclusion criteria: Patients with a previous history of coronary artery disease and patients with hypercoagulability, including hereditary and acquired hypercoagulability states such as oral contraceptive use, pregnancy, autoimmune diseases, and hormone replacement therapy, were excluded. Patients with significant renal disease defined as serum creatinine ≥ 140 μmol/L, those with hypothyroidism, and those taking vitamin supplements were excluded.

### 2.4 Data collection

Data on sociodemographic characteristics including disease history, smoking status, diabetes, hypertension, dyslipidemia, family history of early coronary heart disease, and body mass index were obtained. An electrocardiographic study, standard transthoracic examination, basic laboratory tests, determination of CPK, troponin, complete blood count, coagulogram, lipid profile, renal and liver function tests were performed.

### 2.5 Cardiovascular data

Assessment of cardiac damage, disease progression, treatment strategies, echocardiographic findings, coronary angiography, and SYNTAX score assessment were recorded. Coronary angiography was performed and assessed by two independent observers. The number of affected vessels and lesions was determined. The Killip classification was used to describe the clinical severity of heart failure at presentation in patients with ACS. Class I indicates no evidence of heart failure; class II indicates mild-to-moderate heart failure; class III indicates pulmonary edema; and class IV indicates cardiogenic shock (19, 20).

The SYNTAX score II 2020 (SSII-2020) was used to characterize anatomical and clinical risk and to classify patients into low, intermediate, or high SYNTAX score categories (21–23). In this manuscript, SYNTAX score category was treated as an internal angiographic/prognostic outcome within the ACS cohort and not as a measure of ACS onset risk.

### 2.6 Blood samples

During the first 24 h after admission and after an overnight fast where clinically feasible, three blood samples were collected from each participant by venipuncture. Two 5-mL samples were collected in plain tubes and one 5-mL sample was collected in an ethylenediaminetetraacetic acid (EDTA) tube by a certified phlebotomist. Blood samples were collected at KFMMC. All samples were analyzed in the KFMMC laboratory except vitamin B1, which was analyzed at Delta Medical Laboratories.

The EDTA sample was used for complete blood count analysis using an automated blood cell counter (ABBOTT Alinity HQ). Serum and plasma were separated within 1 h of sample collection by centrifugation and aliquots were stored at −80 degrees C until analysis. Homocysteine measurement was performed using the specified laboratory protocol. The authors should ensure that the final submitted version consistently states the exact specimen type used for homocysteine measurement.

The first serum sample was used to assess cholesterol, low-density lipoprotein cholesterol (LDL-C), high-density lipoprotein cholesterol (HDL-C), triglycerides, and cardiac enzymes. The second serum sample was used to assess vitamins B12, B6, B1, HCY, and folate. Vitamin B12 and folate were measured using the ABBOTT Alinity i system. Vitamin B1 was analyzed externally at Delta Medical Laboratories. Vitamin B12 deficiency was defined as < 147.6 pmol/L (< 200 pg/mL), folate deficiency as < 3 ng/mL, vitamin B1 deficiency as < 70 nmol/L, vitamin B6 deficiency as < 20 nmol/L, and hyperhomocysteinemia as HCY > 15 micromol/L. Assay manufacturers, analytical coefficients of variation, reference ranges, and any unit conversions should be verified against laboratory records and reported in the final version.

### 2.7 Statistical analysis

All statistical analyses were performed using JASP (version 0.18), with Python (version 3.x) used for data visualization. Descriptive statistics were computed to summarize the demographic, clinical, laboratory, echocardiographic, and angiographic characteristics of the study cohort. Continuous variables are reported as means with standard deviations or medians with interquartile ranges, as appropriate, and categorical variables are reported as frequencies and percentages. Normality of all continuous variables was assessed using the Shapiro-Wilk test. As the distribution of biomarker variables deviated significantly from normality (*W* = 0.812, *p* < 0.001), nonparametric methods were applied throughout. Associations between homocysteine and B-vitamin and folate levels were evaluated using Spearman's rank-order correlation, with the squared Spearman's rho (ρ^2^) reported as an index of explained variance. A statistically significant negative correlation was observed between homocysteine and folate (ρ = −0.254, *p* = 0.040), while no significant correlations were identified between homocysteine and vitamins B12, B1, or B6.

To evaluate the independent association between selected biomarker and clinical variables and coronary anatomical complexity, an ordinal logistic regression model was fitted with SYNTAX Score II 2020 (SSII-2020) risk category — classified as low, intermediate, or high — as the ordered outcome. This model was constructed to examine predictors of coronary complexity within the acute coronary syndrome (ACS) cohort and was not intended to model the risk of ACS onset or to distinguish between ACS subtypes. Given the modest sample size of *n* = 80 and the limited number of outcome events across SSII-2020 strata (*n* = 6 high-risk; *n* = 8 intermediate-risk), a principled model reduction was undertaken prior to final estimation. Variables with very low event frequencies — specifically, drug abuse (*n* = 2, 2.5%), family history of premature coronary artery disease (*n* = 7, 8.7%), unstable angina as a type of presentation (*n* = 3, 3.75%), female gender (*n* = 5, 6.25%), and aldosterone inhibitor use — were excluded from the final model, as their inclusion produced near-complete separation and implausibly inflated coefficient estimates with disproportionately wide standard errors, reflecting numerical instability rather than true predictor effects. The final model retained eight predictors: vitamins B12, B1, and B6; heart rate; age; diabetes mellitus; hypertension; and smoking status. Model coefficients are reported alongside odds ratios (OR) with 95% confidence intervals (CI), computed as the exponentiated regression coefficients and their corresponding exponentiated confidence bounds, respectively. Statistical significance was defined as *p* ≤ 0.05 (two-tailed) throughout.

### 2.8 Ethics

This research has been conducted in accordance with the Declaration of Helsinki. The Ethical Approval was obtained from Armed Forces Hospitals Eastern Province Institutional Review Board (IRB); AFHER-IRB-REN-2022-004 (ORIGINAL IRB # AFHER-IRB-REN-2021-001).

### 2.9 Consent to participate

All participants provided written informed consent for data collection and publication prior to the study. Participation was voluntary, and participants could withdraw from the study at any time.

## 3 Results

The sample consisted of 80 participants, of which 93.75% were males, and females constituted 6.25% of the sample. Regarding ethnicity, 96.25% of the participants were Arabs. The majority were Saudi nationals (93.75%), while non-Saudis constituted 6.25%.

Descriptive statistics of cardiovascular risk factors showed that 35% of participants were diagnosed with hypertension; diabetes mellitus was present in 41.25% of participants. Similarly, dyslipidemia, another critical risk factor for cardiovascular disease, was present in 41.25% of the sample, and 68.75% of the participants were identified as smokers. In terms of BMI, most of the participants fell into the overweight (40%) and obese (26.25%) categories, and 17.5% were classified as extremely obese. Drug abuse was relatively rare in this cohort, with only 2.5% of participants reporting a history of drug abuse. No history of heart failure was recorded.

The distribution of cardiovascular presentations among the participants showed an almost equal distribution between non-ST-segment elevation myocardial infarction (NSTEMI) at 47.5% and ST-segment elevation myocardial infarction (STEMI) at 48.75%. Furthermore, unstable angina, although less common (3.75%), represented another significant clinical manifestation of acute coronary syndrome in this study sample.

Data on medication use among participants after acute coronary syndromes showed significant adherence to critical treatments needed to maintain cardiovascular health. Aspirin was prescribed to all participants (100%), followed by ticagrelor (52.5%) and clopidogrel (40%), demonstrating different antiplatelet strategies tailored to patient needs. The high rates of beta-blocker use (91.25%) and ACE inhibitors or angiotensin receptor blockers (82.5%) highlighted the need for good control of cardiovascular risk factors, which is critical for long-term heart health. Statin therapy was almost universal (98.75%), highlighting its integral role in lipid management and secondary prevention after ACS. Heparins (UH or LMWH) were used in 77.5% of cases, supplemented by GP 2b/3a inhibitors in 22.5%. This indicates the targeted approaches to thrombotic risk management during acute events. Finally, oral hypoglycemic agents, prescribed in 18.75% of cases, provide glycemic control in patients with diabetes mellitus, supporting the complex therapy of cardiovascular diseases.

Heart rate and blood pressure data showed a mean heart rate of 81.8 bpm with a standard deviation (SD) of 18.773 bpm, ranging from 51 to 160 bpm. Systolic blood pressure (SBP) had a mean of 130.775 mmHg and SD of 23.225 mmHg, ranging from 90 to 220 mmHg. Diastolic blood pressure (DBP) had a mean of 80.313 mmHg and SD of 15.955 mmHg, ranging from 51 to 140 mmHg. The Killip classification showed that 79 patients (98.75%) were class I and 1 patient (1.25%) was class II. Cardiac arrest occurred in 2 cases (2.5%) (Table 1).

**Table 1:** Heart rate, blood pressure, and heart failure analysis data as per the Killip classification (*n* = 80).

Cardiac arrest	Frequency (n)	Percentage (%)
No (0)	78	97.5
Yes (1)	2	2.5
**CHF killip class**		
Class I	79	98.75
Class II	1	1.25
**Heart rate and blood pressure**	**Mean**	**Std. deviation**
HR (bpm)	81.8	18.773
SBP	130.775	23.225
DBP	80.313	15.955

Laboratory results provided information on biomarkers and blood parameters relevant to cardiovascular status among study participants. Troponin results were reported as positive in 65% of tests, negative in 3.75%, and very high in 1.25%; assay-specific thresholds for these categories should be stated in the final version. NT-proBNP testing was performed in 83.75% of participants. The mean troponin level was 161.730 pg/mL, with a wide range (12.500 to 1,031.500 pg/mL), indicating variability in myocardial injury severity. Participants had a mean nadir hemoglobin level of 14.183 g/dL. The lipid profile showed mean LDL-C of 3.630 mmol/L, triglycerides of 1.971 mmol/L, and HDL-C of 1.010 mmol/L. Mean HCY was 9.152 micromol/L, which is below the predefined hyperhomocysteinemia threshold of >15 micromol/L; therefore, the proportion of participants above this threshold should be explicitly reported once verified from the dataset. Mean levels of vitamin B12, folate, vitamin B1, and vitamin B6 were 270.653 pg/mL, 21.812 ng/mL, 27.616 ng/mL, and 12.218 ng/mL, respectively.

Table 2 summarizes the use of ICA and interventional procedures among study participants with acute coronary syndromes. The high rates of coronary angiography (97.5%) and percutaneous coronary intervention (PCI) (62.5%) reflect standard practice in the diagnosis and treatment of coronary artery disease. Thrombectomy devices were used in 10% of cases, indicating targeted management of thrombotic complications during PCI. The distribution of SYNTAX scores highlights the complexity of the treated coronary lesions, with the majority of participants having low scores (82.5%), indicating fewer complex lesions, while a smaller proportion had intermediate (10%) or high scores (7.5%), reflecting more complex cases requiring advanced intervention strategies (Table 2).

**Table 2:** Data on invasive coronary angiography (ICA) and SYNTAX score.

Variable		Yes *n* (%)	No *n* (%)
**Coronary angiography done**		78 (97.5)	2 (2.5)
**PCI done**		50 (62.5)	30 (37.5)
**Thrombectomy device used**		8 (10)	72 (90)
**PCI to left main stem**		2 (2.5)	78 (97.5)
**PCI to LAD (left anterior descending coronary artery)**		29 (36.25)	51 (63.75)
**PCI to LCX (left circumflex coronary artery)**		8 (10)	72 (90)
**PCI to RCA (right coronary artery)**		15 (18.75)	65 (81.25)
**PCI to branches**		9 (11.25)	71 (88.75)
**SYNTAX score**	**High** ***n*** **(%)**	**Intermediate** ***n*** **(%)**	**Low** ***n*** **(%)**
	6 (7.5)	8 (10)	66 (82.5)

Echocardiographic results showed that a significant proportion of patients demonstrated normal left ventricular systolic function (51.25%), indicating preserved cardiac function after the event. Mild left ventricular systolic dysfunction was noted in 30% of cases, indicating a slight deterioration in cardiac pump efficiency (Table 3). Fewer patients showed moderate (8.75%) and severe (8.75%) left ventricular systolic dysfunction, indicating more pronounced impairment requiring closer monitoring and potentially more intensive treatment strategies. Regarding hospitalization outcomes, it was found that no participants had a recurrent myocardial infarction (MI) during their hospital stay. The mean length of hospital stay was approximately 6.3 days (SD 5.152 days). The range was from 1 to 26 days, highlighting the different recovery trajectories among patients (Table 3).

**Table 3:** Descriptive statistics of echocardiographic findings and hospitalization data (*n* = 80).

Variables	***N*** **(%)**			
Mild LV systolic dysfunction	24 (30)			
Moderate LV syst dysfunction	7 (8.75)			
Normal LV systolic function	41 (51.25)			
Severe LV systolic dysfunction	7 (8.75)			
No recurrent myocardial infarction	80 (100)			
Hospital stays (days)	**Mean**	**SD**	**MIN**	**MAX**
	6.3	5.152	1	26

Spearman correlation analysis showed a weak but statistically significant negative association between HCY and folate (rho = −0.254, *p* = 0.040; approximate R2 = 0.065), suggesting that only a small proportion of variability in HCY was explained by folate levels in this cohort. No statistically significant correlations were observed between HCY and vitamin B12 (rho = −0.207, *p* = 0.098; approximate R2 = 0.043), vitamin B1 (rho = −0.070, *p* = 0.617; approximate R2 = 0.005), or vitamin B6 (rho = −0.077, *p* = 0.585; approximate R2 = 0.006). The Shapiro-Wilk test showed significant deviation from normality (W = 0.812, *p* < 0.001), supporting the use of non-parametric correlation analysis (Table 4).

**Table 4:** Spearman correlations between biomarkers and homocysteine and hypothesis testing.

	Spearman's rho		*p*-value
**HOMOCYSTEINE**	B12	−0.207	0.098
	Folate	−0.254	0.04^*^
	B1	−0.07	0.617
	B6	−0.077	0.585
**Shapiro-Wilk**	0.812		0.001^*^

^*^ *p* < 0.05.

An exploratory ordinal logistic regression analysis was performed to assess associations between selected biomarkers/clinical variables and SYNTAX score category (low, intermediate, high) within the premature ACS cohort (Table 5). The model demonstrated improved fit compared with the null model (deviance decreased from 69.291 to 15.401; chi-square = 53.890, *p* < 0.001). However, given the small sample size and sparse categories, these estimates should be interpreted as exploratory and potentially unstable. The model should not be interpreted as predicting ACS onset or demonstrating protective or pathogenic biomarker effects. In the final statistical table, odds ratios and 95% confidence intervals should be provided, and the model should be reviewed by a statistician to reduce sparse predictors and minimize overfitting.

**Table 5:** Exploratory ordinal logistic regression assessing associations between selected biomarkers/clinical variables and SYNTAX score category within patients with premature ACS.

					95% CI (Coefficients)			95% CI (OR)	
	Estimate	Std. Error	z	p	Lower	Upper	OR	Lower	Upper
(Intercept) ⋆ 1	−8.321	5.094	−1.634	0.102	−18.304	1.663	—	—	—
(Intercept) ⋆ 2	−7.105	5.045	−1.409	0.159	−16.992	2.782	—	—	—
B12	−0.001	0.004	−0.306	0.760	−0.008	0.006	0.9990	0.9920	1.0060
B1	0.154	0.115	1.334	0.182	−0.072	0.380	1.1665	0.9305	1.4623
B6	−0.125	0.157	−0.792	0.428	−0.433	0.184	0.8825	0.6486	1.2020
HR (bpm)	0.008	0.022	0.375	0.708	−0.034	0.050	1.0080	0.9666	1.0513
Age	0.062	0.082	0.750	0.453	−0.099	0.222	1.0640	0.9057	1.2486
Diabetes mellitus	−2.389	1.039	−2.299	0.021	−4.425	−0.352	0.0917	0.0120	0.7033
Hypertension	2.382	1.017	2.342	0.019	0.389	4.376	10.827	1.4755	79.519
Smoking status	−0.439	0.838	−0.524	0.601	−2.082	1.204	0.6447	0.1247	3.3334

Odds ratios and 95% confidence intervals should be added after statistical re-analysis or confirmation of the final ordinal regression model. Current coefficients should be interpreted cautiously because of sparse categories and potential overfitting. SCORE (High-Reference) levels: 1:HIGH, 1:INTERMEDIATE, 1:LOW. Linear predictors: logitlink (P[Y ≤ 1]), logitlink (P[Y ≤ 2]). OR, odds ratio (exp[estimate]). CI, confidence interval. Intercept threshold parameters do not have meaningful OR interpretations and are denoted —. Highlighted rows indicate *p* < 0.05.

Prior to interpreting the final ordinal logistic regression model, a principled reduction of predictors was undertaken to address concerns related to data sparsity, quasi-complete separation, and model overfitting. Given the modest sample size of *n* = 80, with only 6 participants classified as high-risk and 8 as intermediate-risk on the SYNTAX Score II 2020 (SSII-2020), the effective number of outcome events available for model estimation was limited to 14 — well below the recommended threshold of 10 to 15 events per predictor variable advocated in the regression modeling literature (24, 25). Consequently, the original 14-predictor model exhibited hallmark signs of overfitting: several coefficients were implausibly large with disproportionately wide standard errors, and the associated odds ratios were astronomically inflated, rendering their clinical interpretation unreliable. Five variables were systematically excluded from the final model based on the criteria detailed below.

### 3.1 Drug Abuse

Drug abuse was reported by only 2 of 80 participants (2.5%), representing an event frequency far too low to support stable parameter estimation in any regression framework. The original model yielded a coefficient of 14.428 (SE = 6.720, *p* = 0.032), which, when exponentiated, produces an odds ratio of approximately 1.84 × 106 — a value that is statistically and clinically implausible. Such extreme estimates are a well-documented consequence of quasi-complete separation, a condition that arises when a predictor nearly perfectly discriminates between outcome categories, causing the maximum likelihood estimator to diverge (60, 62). Although the associated *p*-value appeared nominally significant, this should not be interpreted as evidence of a true effect; rather, it reflects the numerical instability inherent in the estimation process when cell counts are near zero. The prevalence of drug abuse observed in the current cohort (2.5%) is also consistent with prior data from the Saudi population, which has documented low rates of substance abuse among patients with premature acute coronary syndrome (ACS) (26). Accordingly, drug abuse was excluded from the final model.

### 3.2 Family history of premature coronary artery disease

Family history of premature coronary artery disease (CAD) was present in only 7 participants (8.7%). The original model estimated a coefficient of 11.461 (SE = 4.510, *p* = 0.011), again producing a numerically unstable odds ratio exceeding 86,000 with confidence intervals spanning several orders of magnitude. This pattern is consistent with separation rather than a genuine, estimable effect. While a positive family history is an established and biologically plausible risk factor for premature CAD (2, 5), the low event frequency in the current dataset precluded its reliable inclusion in the multivariable model. Notably, the low prevalence of family history observed here (8.7%) is consistent with the reported genetic predisposition rates in Gulf-region PCAD cohorts, where environmental and lifestyle risk factors tend to predominate (3, 26). Investigators in future studies with larger sample sizes will be better positioned to quantify the independent contribution of family history to SYNTAX score severity.

### 3.3 Type of presentation: unstable angina

Unstable angina was the least frequent type of ACS presentation in this cohort, occurring in only 3 participants (3.75%). The original model produced a coefficient of 19.625 (SE = 7.882, *p* = 0.013) for this category, representing the most extreme instance of separation-driven inflation in the full model. As the reference category for the “Type of Presentation” variable was NSTEMI, the near-complete absence of cases in the unstable angina cell rendered any comparative estimate computationally unreliable. Beyond the frequency concern, a conceptual consideration also supports its exclusion: the type of ACS presentation is, to a meaningful extent, an expression of disease severity rather than an antecedent risk factor, and its inclusion as a predictor of SYNTAX score introduces a circularity that may confound interpretation. The “Type of Presentation: STEMI” category (*n* = 39, 48.75%), though adequately represented, was also non-significant in the original model (*p* = 0.323), and the entire “Type of Presentation” variable was therefore removed to preserve model parsimony and avoid conflating predictors with outcome-related constructs.

### 3.4 Gender

The study cohort was predominantly male (93.75%), with only 5 female participants (6.25%), reflecting the well-documented sex disparity in premature ACS, particularly in Middle Eastern populations (3, 26). This severe imbalance in sex distribution precluded stable estimation of a gender effect within the regression model. The original coefficient for gender (male) was −3.750 (SE = 3.702, *p* = 0.311), which was non-significant and associated with a confidence interval that crossed zero by a considerable margin (−11.005 to 3.505), confirming that the model lacked the statistical power to detect any gender-related difference in SYNTAX score risk. Retaining a predictor with this degree of sparsity and non-significance would have consumed a degree of freedom without contributing meaningful information, further exacerbating the overfitting problem. Gender was therefore excluded from the final model. Importantly, this exclusion does not preclude discussion of the sex distribution as a descriptive finding, nor does it diminish its relevance as a known epidemiological characteristic of premature ACS.

### 3.5 Aldosterone inhibitor (Spironolactone)

Spironolactone use was reported in approximately 25% of participants. While this frequency is higher than those of the variables described above, the original model produced a coefficient of 5.902 (SE = 2.444, *p* = 0.016) — a value whose magnitude and SE ratio suggested residual separation within specific SYNTAX score subcategories. Cross-tabulation of spironolactone use against SYNTAX score strata revealed insufficient cell counts to support reliable estimation of this effect, particularly within the high-risk category. Moreover, spironolactone use in this cohort likely reflects a post-diagnosis treatment decision rather than a pre-existing risk exposure, introducing potential endogeneity into the model. Aldosterone inhibitors are prescribed in response to left ventricular dysfunction and elevated aldosterone levels — both of which are downstream consequences of coronary artery disease severity (27, 28). Including a treatment variable whose initiation may be determined by the very outcome it is modeled to predict creates a temporal and causal ambiguity that is difficult to resolve in a cross-sectional design. For these methodological reasons, spironolactone use was excluded from the final model.

### 3.6 Final refined model

Following the exclusion of the five variables described above, the final ordinal logistic regression model retained eight predictors: vitamins B12, B1, and B6 (the primary biomarkers of interest); heart rate (bpm) and age (continuous clinical covariates); and diabetes mellitus, hypertension, and smoking status (binary cardiovascular risk factors with sufficient event frequencies). This reduced predictor set aligns with the 10-events-per-variable guideline, yielding a substantially more stable parameter estimation. The refined model exhibited markedly attenuated coefficients with more proportionate standard errors, and the disappearance of separation-driven inflation confirms that the original instability was attributable to sparse predictors rather than a genuine signal. The final model thus provides a more defensible basis for clinical inference regarding the association of B-vitamin levels and established cardiovascular risk factors with SYNTAX score-defined coronary complexity in patients with premature ACS.

The final ordinal logistic regression model, fitted with eight predictors against SYNTAX Score II 2020 (SSII-2020) risk category as the ordered outcome (Low, Intermediate, High), demonstrated adequate overall fit compared to the null model (χ^2^ = 53.89, *p* < .001). Among the biomarkers of primary interest, vitamins B12, B1, and B6 did not reach statistical significance in the final model (*p* = 0.760, 0.182, and 0.428, respectively), with odds ratios approximating unity [OR = 0.999, 95% CI (0.992, 1.006); OR = 1.167, 95% CI (0.931, 1.462); OR = 0.883, 95% CI (0.649, 1.202)], suggesting that, after adjustment for cardiovascular risk factors, these B-vitamin levels were not independently associated with coronary complexity as quantified by the SSII-2020 in this cohort. Similarly, heart rate [OR = 1.008, 95% CI (0.967, 1.051), *p* = 0.708], age [OR = 1.064, 95% CI (0.906, 1.249), *p* = 0.453], and smoking status [OR = 0.645, 95% CI (0.125, 3.333), *p* = 0.601] were likewise non-significant. In contrast, two cardiovascular comorbidities emerged as statistically significant independent predictors of SSII-2020 risk category. Diabetes mellitus was associated with significantly lower odds of being classified in a higher-risk SSII-2020 category [OR = 0.092, 95% CI (0.012, 0.703), *p* = 0.021]. Conversely, hypertension was associated with significantly greater odds of a higher-risk SSII-2020 classification [OR = 10.827, 95% CI (1.476, 79.519), *p* = 0.019]. The wide confidence interval for hypertension (1.476 to 79.519) warrants interpretive caution and reflects the limited statistical power inherent to the modest sample size; nonetheless, the direction and magnitude of the effect are clinically plausible and consistent with prior evidence. Collectively, these findings underscore the predominant role of established hemodynamic risk factors particularly hypertension over B-vitamin nutritional status in determining coronary anatomical complexity among patients presenting with premature acute coronary syndrome in this cohort.

**Figure 1:**
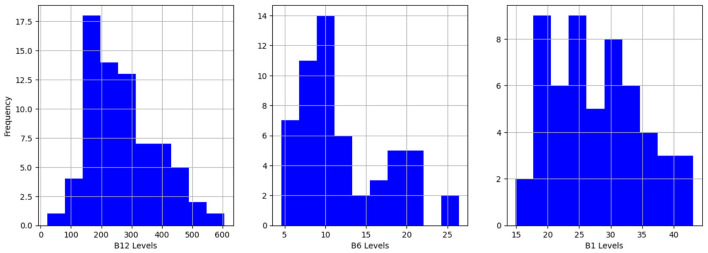
Visualization of laboratory workup of B12, B6, B1 levels.

The distribution of B12 levels was skewed to the right, with most values concentrated between 100 and 300 pg/mL and a peak around 150–200 pg/mL (Figure 1). B1 levels showed a relatively even distribution across the observed range, whereas B6 levels were concentrated in the lower range. These figures should be revised in the final submission to include complete axis labels, units, and high-resolution formatting according to journal requirements.

**Figure 2:**
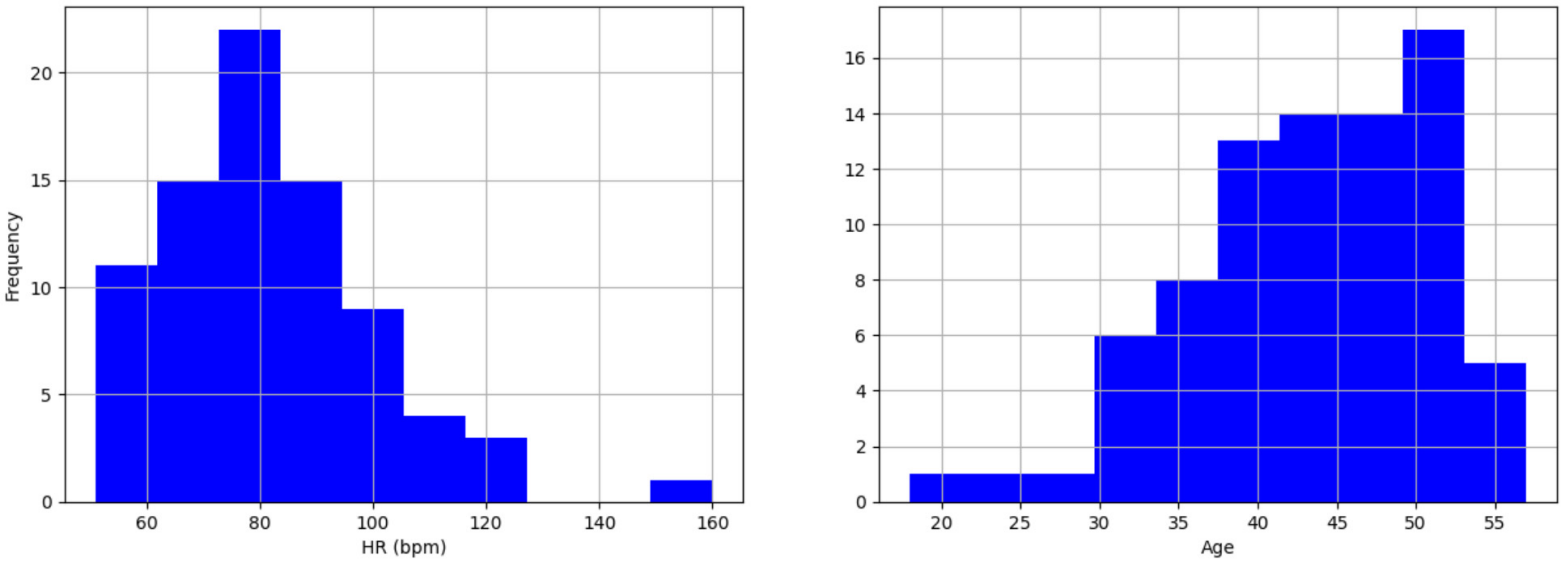
The distribution of heart beats per minute and age groups.

Heart rate followed an approximately normal distribution centered around 80 bpm, with a few higher values extending to 160 bpm. Age distribution was right-skewed, with most participants between 30 and 55 years (Figure 2). Figure axes, units, and legends should be checked and standardized before resubmission.

## 4 Discussion

To the authors' knowledge, this is among the first Saudi studies to describe HCY, vitamin B1, B6, B12, and folate profiles in patients presenting with premature ACS. The principal finding was a weak inverse association between HCY and folate, whereas HCY was not significantly correlated with vitamins B12, B1, or B6. Because the study included only patients with established ACS and had no non-ACS comparator group, the findings should be interpreted as within-cohort associations rather than evidence of causality, ACS risk prediction, or protective/pathogenic biomarker effects.

The cohort was predominantly male and Saudi, reflecting the recruitment pattern of this single-center sample. This marked sex imbalance limits generalizability, particularly to female patients, and prevents meaningful sex-specific analyses. The findings should therefore be interpreted primarily in the context of a mostly male premature ACS cohort.

Traditional cardiovascular risk factors were common in this cohort, including smoking, diabetes, dyslipidemia, hypertension, and elevated body mass index. These observations are consistent with regional and international reports describing a high burden of modifiable risk factors among patients with premature CAD (29–36). However, the present design does not allow comparison with the background population or determination of which factors contributed to ACS onset.

The weak inverse relationship between HCY and folate is biologically plausible because folate participates in homocysteine remethylation. Nevertheless, the effect size was small, and the mean HCY level was below the predefined hyperhomocysteinemia threshold. Therefore, statements suggesting that most participants had elevated HCY should be avoided unless the exact number and percentage above 15 micromol/L are confirmed from the dataset.

The absence of significant correlations between HCY and vitamins B12, B1, and B6 may reflect several factors, including limited sample size, acute-phase metabolic changes during ACS, individual variability in vitamin metabolism, renal and hepatic function, unmeasured dietary intake, and genetic determinants such as MTHFR polymorphisms. These factors are particularly relevant because blood sampling occurred within 24 h of ACS admission and dietary/genetic data were not collected.

The exploratory regression analysis was reframed as an analysis of SYNTAX score category rather than ACS onset or ACS subtype. This distinction is important because all participants already had ACS. The regression results may generate hypotheses regarding associations between biomarker/clinical variables and coronary lesion complexity, but they should not be used to infer that a biomarker protects against ACS or increases ACS risk.

The original regression model included multiple predictors despite a small sample size and sparse categories, which increases the risk of overfitting, unstable estimates, sparse-data bias, and type I error. Large coefficients for low-frequency variables should therefore be interpreted with caution. The final model should be simplified based on clinical relevance and data distribution, with odds ratios, 95% confidence intervals, and model diagnostics reported.

Angiographic and echocardiographic findings showed that most patients had low SYNTAX score categories and preserved or mildly impaired left ventricular systolic function. These data help characterize the clinical profile of this cohort, but they should not be overinterpreted beyond descriptive relevance.

Overall, the study contributes preliminary regional data on HCY and B-vitamin profiles in premature ACS. Its main value is descriptive and hypothesis-generating. Larger multicenter studies with matched controls, standardized dietary assessment, genetic testing, and longitudinal follow-up are required to determine whether these biomarkers have diagnostic, prognostic, or therapeutic relevance in Saudi and Middle Eastern populations.

## 5 Limitations

This study has several important limitations. First, the sample size was relatively small (n=80) and recruitment was limited to a single center due to COVID-19 restrictions, reducing statistical power and generalizability. Second, the cross-sectional single-cohort design, without healthy controls, non-ACS comparators, or older ACS comparators, precludes causal inference and prevents assessment of biomarker associations with ACS onset. Third, the study population was predominantly male and Saudi, limiting applicability to women and more diverse populations. Fourth, dietary intake of folate and B vitamins, supplement history beyond exclusion criteria, and genetic determinants such as MTHFR polymorphisms were not assessed. Fifth, blood sampling within 24 h of ACS admission may have been influenced by acute-phase metabolic changes. Finally, the exploratory regression model is limited by the small sample size and sparse categories; therefore, regression estimates should be considered hypothesis-generating and require validation in larger cohorts.

## 6 Conclusion

This single-center exploratory study describes HCY, B-vitamin, and folate profiles in Saudi patients with premature ACS. A weak inverse association was observed between HCY and folate, while no significant correlations were observed between HCY and vitamins B12, B1, or B6. Because the study lacked a control group and included only patients with established ACS, the findings should not be interpreted as evidence of ACS risk reduction, causality, or protective/pathogenic biomarker effects. These preliminary data support the need for larger controlled studies incorporating dietary assessment, genetic testing, standardized biomarker measurement, and longitudinal clinical outcomes.

## Data Availability

The original contributions presented in the study are included in the article/supplementary material, further inquiries can be directed to the corresponding author/s.
